# Strategies to Overcome HLA Sensitization and Improve Access to Retransplantation after Kidney Graft Loss

**DOI:** 10.3390/jcm11195753

**Published:** 2022-09-28

**Authors:** Rita Leal, Clara Pardinhas, António Martinho, Helena Oliveira Sá, Arnaldo Figueiredo, Rui Alves

**Affiliations:** 1 Nephrology Department, Centro Hospitalar e Universitário de Coimbra, 3000-548 Coimbra, Portugal; 2 Faculty of Medicine, University of Coimbra, 3004-531 Coimbra, Portugal; 3 Coimbra Histocompatibility Center, Portuguese Institute of Blood and Transplantation, 3041-861 Coimbra, Portugal; 4 Urology and Kidney Transplantation Unit, Centro Hospitalar e Universitário de Coimbra, 3000-548 Coimbra, Portugal

**Keywords:** allossensitization, allocation, immunosuppression, kidney graft failure, kidney retransplantation

## Abstract

An increasing number of patients waitlisted for kidney transplantation have a previously failed graft. Retransplantation provides a significant improvement in morbidity, mortality, and quality of life when compared to dialysis. However, HLA sensitization is a major barrier to kidney retransplantation and the majority of the highly sensitized patients are waiting for a subsequent kidney transplant. A multidisciplinary team that includes immunogeneticists, transplant nephrologists and surgeons, and adequate allocation policies is fundamental to increase access to a kidney retransplant. A review of Pubmed, ScienceDirect, and the Cochrane Library was performed on the challenges of kidney retransplantation after graft loss, focusing on the HLA barrier and new strategies to overcome sensitization. Conclusion: Technical advances in immunogenetics, new desensitization protocols, and complex allocation programs have emerged in recent years to provide a new hope to kidney recipients with a previously failed graft.

## 1. Introduction

There is an increasing number of kidney transplant (KT) recipients with graft loss returning to dialysis, and patients waiting for a subsequent KT represent a significant portion of the waiting list [1,2].

For eligible patients, retransplantation offers the largest survival benefit, even in challenging subgroups such as diabetics or the elderly [3,4]. Unfortunately, the access to a subsequent KT is frequently compromised by HLA sensitization and patients waiting for a retransplant represent the majority of the highly sensitized patients waitlisted [5,6]. This is a major concern especially for young chronic kidney disease (CKD) patients, that are more prone to need more than one KT during their lifetime, and are futile waiting for an HLA compatible donor [7,8].

Recent immunogenetic technical advances have allowed a better characterization of the HLA sensitization profile of patients waiting for a KT, and improved our knowledge on the dynamics and risk factors for allosensitization [9,10]. This has led to more awareness on sensitization prevention including better HLA matching, caution with sensitizing events, strategies to increase IS immunosuppression (IS) adherence and concerns on IS withdrawal after first graft loss [11]. New immunosuppressive drugs that effectively decrease HLA antibodies before KT are emerging with promising results [12]. Multinational allocation systems, focused on the highly sensitized, were developed with an impressive and immediate decrease of these patients on the waiting list [13,14].

Several groups are focusing on the management of the failing graft and strategies to increase the transplant success on the sensitized patients are highlights in the KT community, with new recommendations and guidelines being published [15,16].

In the current review we explore the benefits of kidney retransplantation, the challenges of HLA sensitization and the newest strategies to increase the access of a subsequent KT for the highly sensitized patients with a previous kidney graft.

## 2. Materials and Methods

From January to July 2022 the authors have performed an extensive literature research from the following databases: NCBI Pubmed, ScienceDirect, the Cochrane Library and the websites clinicaltrails.org and uptodate.com. Full articles were accessed and reviewed. For the included articles, we used the tools “reference lists” and “related articles” of PubMed to increase our search. We used MeSH terms and free text, according to the specific chapter and in different combinations. The main keywords used were “acute rejection”, “acceptable mismatch”, “allocation”, “antibody mediated rejection”, “desensitization”, “epitope”, “Eurotransplant”, “failing allograft”, “highly sensitized”, “HLA”, “imlifidase”, “immunosuppression”, “immunogenetics”, “kidney transplantation”, “living donation”, “mismatch”, “paired donation”, “panel reactive antibody”, “retransplantation”, “sensitization”. There were no restrictions on publication date, but articles published in the last five-years were prioritized. Only articles in English were selected.

## 3. The Benefits of Retransplantation

KT recipients returning to dialysis have globally worst outcomes including higher mortality rates, higher risk of hospitalization, lower quality of life scores and a higher burden of depression [17,18,19,20]. Long-term IS, a chronic inflammatory state, previous infection and suboptimal CKD management are important contributors to worst outcomes in this population [21]. Retransplantation offers the largest survival benefit to patients with failed transplants, with mortality rate reduction ranging from 20% to 88%, depending on specific comorbidities and transplant era [4,22,23]. A recent cohort study using large USRDS data evaluated the relative risk of death in second transplant recipients (*n* = 3848) compared to waitlisted transplant-failure patients with equal lengths of time since waitlisting (*n* = 12021). Retransplantation was associated with a 68% lower adjusted risk of death compared with dialysis and this benefit was observed in all subgroups examined including diabetic patients, patients older than 60 years-old, and patients with PRA > 80%. [3] The survival benefit of retransplantation has been addressed in other specific subgroups including patients with previous posttransplant lymphoproliferative disorders [24], first graft loss due to BK nephropathy [25], third or fourth kidney retransplant [26], and older recipients [27,28] and found similar graft and patient survival rates. Even in patients older than 70 years-old, there was a slight survival advantage in retransplantation as opposed to remaining on dialysis after first graft failure [29]. Similarly to first graft recipients, only the first month post retransplant increases the risk of mortality compared to dialysis [4]. Large cohort studies have showed inferior graft outcomes of subsequent kidney transplants, including more episodes of acute rejection or primary graft dysfunction but the benefits on patient survival are maintained [3,30]. 

Considering the benefits of retransplantation, the 2014 British Transplant Society Guidelines for the Management of the Failing Kidney Transplant, recommend that patients suitable for retransplant should be evaluated when graft failure is anticipated within one year, and ideally provide preemptive retransplantation [31]. Timely relisting is particularly important since recently published data showed that the survival benefit of retransplantation diminishes with longer waitlist times [32,33].

## 4. The HLA Barrier and Sensitization

The most common sensitizing events, by growing potential, are blood transfusions, pregnancy, and solid organ transplantation [34,35]. Redfield et al., analyzed the impact of different sensitization events in a group of highly sensitized patients and found that transfusion and pregnancy were the sensitizing event only in 5 and 20%, respectively, and that the risk of graft loss was significantly higher in patients with a prior KT [34]. Several other cohorts have shown that patients waiting for retransplantation have significantly higher rates of HLA sensitization and represent the majority of the highly sensitized patients on the waiting list [5,6]. A thorough assessment of HLA sensitization on patients relisted for KT is determinant to increase the chances of finding a compatible donor and improve retransplantation outcomes. 

Solid-phase assays have been developed in the last decade and represent a major advance in HLA study of KT recipients. These assays are highly sensitive in detecting donor specific antibodies (DSA), can quantify their strength measured by mean fluorescence intensity (MFI), and define the unacceptable HLA antigens for each KT candidate [36]. They also allowed the development of calculated PRA (cPRA) and introduced the virtual crossmatch [9,37]. Despite their unquestionable value, solid phase assays have limitations. The definition of unacceptable antigen varies across different transplant centers, false positive results can occur and clinically irrelevant HLA antibodies are frequently considered [6,38]. Gombos et al. recently reported that 62.5% of prospective kidney recipients with no antibodies by complement-dependent cytotoxicity or enzyme-linked immunosorbent assays had at least one HLA antibody detectable with solid-phase assays and an important percentage of patients without a history of sensitization had detectable HLA antibodies with MFI values > 14,000. If these antibodies are directed against an highly frequent antigen, it extremely restricts the chance of finding a donor [39]. The 2022 European Guideline for the Management of Kidney Transplant Patients with HLA Antibodies recommends that defining unacceptable mismatches in highly sensitized patients on the base of weak antibody reactivity in solid-phase assays must be cautious and weight the poorly defined risk of antibody-mediated versus prolonged waiting time and associated mortality and morbidity [16].

Immunogenetics and epitope analysis is gaining importance in the majority of the transplant centers. Anti-HLA antibodies do not restrictively recognize a single whole HLA antigen, instead they recognize short polymorphic sequences of amino acids in antibody accessible positions, called epitopes. These epitopes are frequently shared by other HLA-antigens which explains why one HLA-antibody will cross-react with several antigens [40]. Several programs have been developed to performed donor-recipient matching on the epitope level, such as the HLAMatchmaker [41]. Epitope matching might overcome several barriers of retransplantation by offering a more precise assessment of donor-recipient HLA compatibility and improving the pool of acceptable antigens and potential donors [42]. 

Cellular immunity also plays an important role in sensitization in patients with a previous KT [43]. Memory B cells are generated after the exposure to non-self HLA, persist for long periods in the bloodstream and secondary lymphoid organs and can be present in the absence of DSA, especially after desensitization protocols [44,45]. The clinical relevance of this cells is yet to be determined but they probably contribute to the development of the novo DSA upon antigen re-exposure [46]. The contribution of donor specific memory B-cells in patients waiting for a subsequent KT is extremely important since it can influence the choice of IS, perhaps including B cell depleting agents.

Despite all the advances in characterizing the immunogenetic profile of a potential KT recipient, the presence or absence of preformed DSA do not characterize with absolute certainty patients’ immunological risk. The group ENGAGE has recently propose a strategy to stratify the humoral risk of candidates to organ transplantation in 5 categories (category 1—the highest risk). Of notice, patients with a previous KT, even without DSA, are never on the lower risk category, considering their potential cellular memory [43].

## 5. Strategies to Increase Retransplantation Access (Figure 1)

### 5.1. Improve HLA Matching in First Kidney Graft

The importance of first graft HLA matching on second KT clinical outcomes is related to the risk of allosensitization and the potential harmful effects of repeated mismatches. 

**Figure 1:**
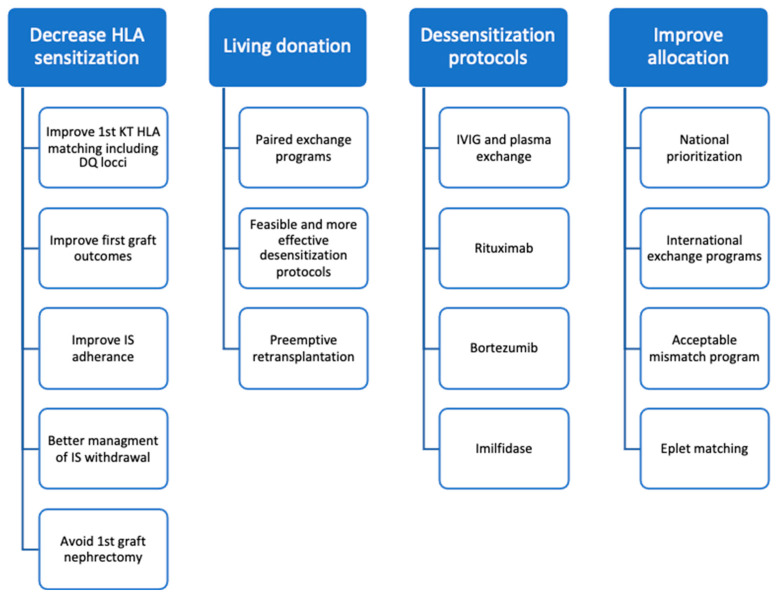
Strategies to increase retransplantation access. KT—kidney transplant; IS—immunosuppression.

A large United Stated registry analysis of 15,980 patients relisted to KT after graft loss have showed that patients with higher HLA-A, -B, and -DR mismatching had greater likelihood to be newly or increasingly sensitized at relisting. Detailed analysis revealed that the -DR locus had only a marginal effect on sensitization, and HLA-A mismatches were associated with steeper increases in PRA [8]. A more recent cohort study, that used single antigen-bead technology, found that HLA class I and II mismatches (including -C and -DQ) on the first KT were independent factors for sensitization on the waiting list (adjusted OR of 1.4 per mismatch) and all loci contributed to the development of HLA antibodies. Singh et al., have studied the impact of first KT eplet mismatch on sensitization trends in 66 patients with vPRA < 10% at relisting and found that IS withdrawal and DQB1 eplet mismatch were independent risk factors for becoming highly [47]. It is noteworthy, that these results suggest an important role for –DQ mismatch in sensitization, however this locus is not considered in the majority of the allocation algorithms. Also, HLA-DQ antibodies are the most common de novo DSA and increase the risk of antibody mediated rejection, transplant glomerulopathy and allograft loss [48,49]. 

The relevance of repeated mismatch remains a controversial topic on retransplantation. Re-exposure to a new load of HLA antigens that were present on a previous donor may increase the risk of acute rejection through the stimulation of memory T cells [50,51]. Studies published before 2000, when IS was less efficient and laboratorial techniques were less advanced, found contradictory results regarding the impact of repeated mismatches on retransplantation outcomes [52,53,54,55]. A recent, large cohort study that included 13,789 retransplanted patients, of which 3868 had at least one repeated mismatch, found no differences in allograft failure. However, a subgroup analysis showed that any class 2 repeated mismatch increased the risk of death-censored graft loss, especially for patients with pretransplant cPRA > 0% or first graft nephrectomy [51]. A previous report on 233 retransplanted patients for which repeated mismatch were allowed when no DSA were present, found that HLA-DR repeated mismatching was a major risk factor for graft loss [56]. It is of utmost importance to clarify the real impact of repeated mismatches in the absence of DSA, including -DQ loci, since avoiding repeated mismatching decreases the pool of donors and consequently increases the waiting time for a subsequent graft.

### 5.2. Immunosuppression Withdrawal and Graft Nephrectomy after Graft Loss

Several authors have addressed the impact of IS withdrawal and graft nephrectomy after graft loss in HLA sensitization, but results are conflicting. The first large observation study, analyzed 119 graft failure with a PRA < 20% at dialysis restart and concluded that weaning IS was an independent predictor of HLA sensitization [57]. A cohort that included 131 KT patients on the waiting list for retransplant showed that maintaining IS with two drugs significantly decreased the risk of developing HLA antibodies, determined by Luminex single-bead assays. Another retrospective study published in 2021, compared IS weaning in three timings: <90 days, 90–180 days and >180 days, and concluded that prolonged IS withdrawal did not reduce sensitization or improved retransplantation rates but decreased the chance of graft nephrectomy [58]. The first prospective multicenter study on IS withdrawal after graft loss was recently published and included 269 patients. Outcomes were compared between two groups: group 1—continuation of IS with CNI and/or antiproliferative and/or prednisone versus group 2: discontinuation of all IS or use of prednisone only. Although numerically lower, there were no statistically significant differences in cPRA or graft intolerance syndrome (GIS) [59]. Maintaining IS after graft has been associated with higher infectious risk [60] and neoplasia [61] but no differences in hospitalization or mortality were found in recent prospective analysis [59].

**Figure 2:**
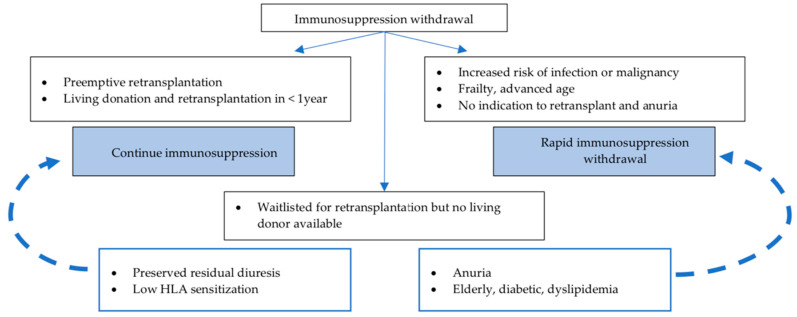
Suggested approach to immunosuppression withdrawal after graft loss. For patients waitlisted for retransplantation without an available living donor, the tendency (dashed arrows) to continue or withdrawal immunosuppresion, should be defined on an individual basis, according to residual diuresis, actual HLA sensitization and other comorbidities.

Figure 2 summarizes a suggestive approach to IS withdrawal after graft loss considering the most recent recommendations.

The British Transplantation Society Guidelines for the Management of the Failing Kidney Transplant recommend that IS should be maintained if there is the prospect of preemptive retransplantation or within one year of starting dialysis. Otherwise, IS must be considered on an individual basis [31]. The Kidney Recipient with Allograft Failure Transition of Care (KRAFT) group consider that patients with a prospect of a subsequent KT, should maintain IS in the first year post graft failure with a reduction of 50% of antimetabolite at dialysis start and suspension at 3 months, and maintaining CNI (with progressive dose reduction) with or without low dose steroids for one-year after graft loss [62]. Must authors are consensual that frequent HLA monitoring should be done, especially when IS is altered or acute events occur, such as infections or signs of GIS [15,31]. In case GIS occurs, recommendation is to start pulse steroids, followed by a slow taper over 6 months and, if no response to steroids occur, propose graft nephrectomy [15].

In most cohorts, HLA sensitization is higher in patients who perform allograft nephrectomy, including studies using single-antigen assays [63,64,65]. However, it is important to interpret these results considering multiple confounding factors. In many cases, graft nephrectomy occurs in the context of GIS after IS withdrawal, which might be the major sensitizing event.

### 5.3. Desensitization Protocols

**Figure 3:**
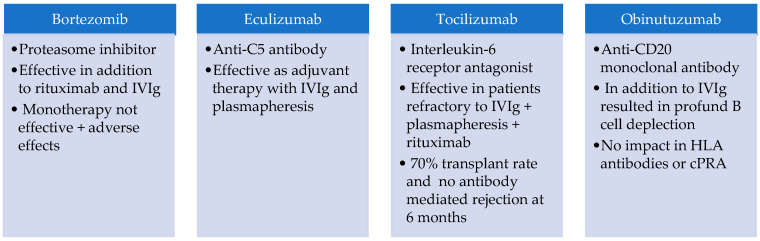
New drugs with potential benefits on dessensitization on small RCT [77,78,79,80,81,82].

The pioneer research in desensitization started with intravenous immunoglobulin (IVIg), that showed the capability to inhibit lymphocyte toxicity in vitro, decrease HLA antibodies in vivo, and increase the rates of KT in highly sensitized patients [66,67,68]. Afterwards, plasmapheresis and immunoadsorption protocols were introduced and showed more effective DSA removal than IVIg alone [69,70]. The addition of anti-CD20 rituximab to IVIg and plasmapheresis protocols, has shown promising results [68,71,72,73]. A recent prospective study reported 3-year outcomes in 29 highly sensitized patients who were desensitized with high dose IVIg and one dose of rituximab after transplantation. The study showed a 46% reduction in the strength of DSA at 1 month after transplant that was sustained throughout the 3-year follow-up period for both class I and class II. Patient and graft survival at follow-up were 95% and 90%, and acute rejection only occurred in 4 patients [74]. Despite the promising results, the existing desensitization protocols have important side effects, high cost and a dubious therapeutic benefit, with incomplete removal of DSA and rebound antibody production [75,76]. Alternative strategies are needed and recently, new drugs are arousing with promising results in small RCT (Figure 3).

Imlifidase, a recombinant cysteine protease derived from *Streptococcus pyogenes*, cleaves all four human subclasses of IgG with strict specificity, which suggests an important role in desensitization. In a phase 1–2 clinical trial in 2017, imlifidase was administered to 25 sensitized patients with an HLA-incompatible donor, in adjunctive therapy with rituximab and IVIg. Total IgG and HLA antibodies were eliminated, and transplantation was possible in 24 of 25 patients. Antibody mediated rejection occurred in ten patients in the first 6 months post-transplant, but only one graft loss was documented at follow-up [83]. A subsequent multinational trial included 18 patients with a median cPRA of 99.83% and a positive crossmatch to their donor. After imlifidase was administered, crossmatches were repeated, and the transplant was performed once the first negative crossmatch resulted. Adjunctive therapy with alentuzumab and rituximab plus high dose IVIg was performed. All patients underwent transplantation within 24 h, and no hyperacute rejection was observed. Three patients had DSA rebound and biopsy proven antibody mediated rejection, all of them with a good therapeutic response to plasmapheresis-based therapies. No severe infections were reported, graft survival was 92% (graft loss for non-immunological reasons) and patient survival was 100% at follow up [84]. The rapid elimination of circulating HLA antibodies, 4–6 h after drug administration, is a major advantage for deceased donor donation [85] but an important limitation is the unpredictable DSA rebound and increased risk of antibody mediated rejection. 

Despite the difficulties and hazards in desensitization, recent multicentric retrospective cohorts have shown a significant survival benefit of HLA-incompatible transplantation after desensitization, when compared to remaining on the waiting list, with a nearly doubling survival benefit [86].

### 5.4. Living Donation, Kidney Paired-Donation and Preemptive Retransplantation

Compared to deceased donor transplantation, living donation transplants have higher rates of immediate function and better graft and patient survival [87,88]. In retransplantation, living donor benefits are not so well established, but a recent cohort that included 325 retransplanted patients, of which 104 with a living donor, showed that living donor retransplantation conferred better graft survival and similar outcomes to first-graft recipients [89]. The advantage of living donation in retransplantation, is even more valuable for the sensitized patients: organ-shortage problem is overcome, and it is easier to cross the HLA barrier challenges. Genetically related donors have better HLA matches [90], ischemia-reperfusion injury tends to be lower which decreases the exposure to HLA antigens [91], IS after first graft loss can be maintained in higher doses [31] and desensitization protocols are more effective [92]. Living donation also allows kidney-paired donation for AB0 or HLA incompatible pairs, with similar outcomes to direct living donation [93,94].

Unfortunately, even under optimal conditions less than 15% of the highly sensitized patients are predicted to find a compatible match in exchange paired programs, especially if the recipient is a group 0 and the donor a group A [95]. There are two strategies to increase the chance of a highly sensitized patients to find a compatible pair in a kidney paired donation run: combined desensitization with paired donation and increase the donor-recipient pool with multicentric exchange programs. Multicentric organized groups for kidney paired donation, such as the National Kidney Registry, have recently shown retransplant rates of 25%, with better HLA matching [96]. 

Preemptive transplantation is associated with significantly better graft and patient survival, especially when a living donor is available [97,98]. Preemptive retransplantation adds other benefits that are extremely valuable for graft and patient survival: it is performed under full immunosuppressive treatment and first allograft is not removed, preventing HLA sensitization [99]. Also, the higher mortality rates in dialysis while on the waiting list, are overcome, vascular capital is preserved and the patients gain significant quality of life [33]. 

Several large cohorts have unanimously concluded that preemptive retransplantation is associated with less acute rejection rates, less delayed graft function, better death-censored graft and increased patient survival [100,101,102]. Interestingly, an exception was noticed in patients with first graft survival inferior to one-year, which had a discrepant 34% increased risk of death-censored graft loss [100]. The British Transplantation Society Guidelines suggest that preemptive retransplantation in suitable candidates is the best option for ongoing renal replacement therapy, with the exception of patients with very early graft failure [31]. Despite the potential benefits of preemptive kidney retransplantation this option still remains rare, with rates ranging from 5–15% [102,103]. 

### 5.5. Allocation Programs for the Highly Sensitized Patients

Table 1 summarizes different allocation programs applied for the sensitized patients.

The European Guideline for the Management of Kidney Transplant Patients with HLA Antibodies, published in 2022, recommends an active policy of prioritizing highly sensitized patients for organ transplantation. For patients without an available living donor, prioritization schemes and acceptable mismatch programs are the first options [16]. 

The Eurotransplant group created the Acceptable Mismatch Program in 1989. Instead of focusing on unacceptable antigens, the group operates according to acceptable HLA matching. After extensive laboratory testing, HLA antigens towards which the patient has not made antibodies, are considered acceptable antigens. In a certain way, this program adds new acceptable antigens to the highly sensitized patients own HLA typing, creating an extended HLA phenotype which significantly increases the donors offer. There is a minimal match criteria of two HLA-DR or one HLA-DR plus one HLA-B, and repeated HLA mismatch is not allowed [104]. When a compatible donor is available, there is mandatory shipment of the organ to the sensitized patient center, within the Eurotransplant group. With the implementation of this program, organ offers are made to roughly 80% of the patients’ in these program, more than 1700 patients have received a transplant and waiting list times have significantly decreased [42,105]. Also, more than 30 years after the implementation of this program, long-term results are very encouraging with rejection rates similar to the non-sensitized and excellent graft survival [14,105]. Future direction of this program include class I and II epitope analysis, extended allocation to loci -C and -DQ, reduce the minimal match criteria for selected patients and restrict the inclusion criteria to patients with a chance of receiving a compatible organ lower than 2% [105]. Despite the remarkable results of the AM program, there are still a group of patients that are not able to find a compatible donor within this program. A European wider acceptable mismatch program was proposed—The EUROSTAM program, that includes Eurotransplant, the UK National Health Service Blood and Transplant, Barcelona, and Athens. Using a simulation tool, a recent published article showed that 27% of the long waiting highly sensitized would be transplanted [13]. 

An interesting approach that is unique in the 2019 UK allocation scheme is the “longevity matching”: (1) match the estimated longevity of the recipient with the estimated longevity of the graft; (2) in younger patients, allocation prioritizes minimizing HLA mismatch. This scheme aims to prioritize younger patients for better matched grafts (HLA and longevity), in order to prolong death censored graft survival, minimize HLA sensitization and improve the likelihood of retransplantation [106,107].

The perfect allocation system might not exist and a reasonable one is very complex to achieve, and should include several factors with different weighting factors according to the individual demographical, clinical and immunogenetic characteristics.

## 6. Conclusions

Kidney retransplantation after graft loss is associated with better quality of life and a longer patient survival, and the number of patients waiting for a subsequent KT is steadily increasing. HLA sensitization remains a major barrier to retransplantation and is particularly challenging in young patients. Impressive progresses in immunogenetics, new pharmacological therapies and international collaborations are changing the paradigm of retransplantation, especially for the highly sensitized patients. However, there are many questions that remain unanswered, including the real impact of HLA sensitization on retransplant long-term outcomes, the best IS weaning scheme and how to develop the fairest and most equitable allocation program.

## Figures and Tables

**Table 1:** Special allocation programs for the highly sensitized.

	Highly Sensitized Definition	Strategies to Increase Transplantation in the Highly Sensitized	Consequences
**KAS (2014)**	cPRA ≥ 98%	Sliding scale with extra points from cPRA ≥ 20% Local (cPRA 98%), regional (cPRA 99%) or national (cPRA 100% allocation	Decrease in waiting times, increased rate of highly sensitized patients getting transplants Higher long-ischemia times and more HLA mismatches.
**Eurotransplant Acceptable mismatch program (1989)**	cPRA ≥ 85% (Include only HLA antibodies explained by sensitizing events)	Increase the recipient HLA antigen phenotype Multi-country allocation	Decrease in waiting times, 80% rate of highly sensitized patients getting transplants, similar acute rejection and graft outcomes than non-sensitized
**Scandiatransplant** **Acceptable mismatch program** **(2020)**	cPRA ≥ 80%	Multi-country allocation Acceptable mismatch program (2009) for patients with a transplantability score ≤ 2%; Prioritization for the highly sensitized	Significant increase of transplant rates in the highly sensitized
**UK allocation program** **(2019)**	cRF > 85%	National allocation Top priority (tier A) if cRF ≥ 85%, 10% patients most difficult to match, ≥7 years on the waiting list	Reduction of 50% on the patients waiting for more than 5 years;
**Spain** **PATHI** **(2015)**	cPRA ≥ 98%	National priority allocation program based on virtual cross-match	10–30-fold increase in the chances of finding a donor Significantly increase of transplantability rate
**France** **Authorized Antigen Program**	cPRA ≥ 85% or cPRA > 70% at transplant	National priority to the higly sensitized Authorized antigen program + age matching	Highly sensitized with increased transplant rates

KAS—Kidney Allocation System OPTN; cPRA—calculated PRA; cRF—calculated reaction frequency.

## Abbreviations

BAFF	B cell activating factor
CKD	chronic kidney disease
CNI	calcineurin inhibitor
cPRA	calculated panel reactive antibody
DSA	donor specific antibody
GIS	graft intolerance syndrome
IS	immunosuppression
IVIg	intravenous immunoglobulin (IVIg)
KAS	Kidney Allocation System
KT	kidney transplant
MFI	mean fluorescence intensity
PRA	panel reactive antibody
